# Probing the Peculiarity of *Eh*RabX10, a pseudoRab GTPase, from the Enteric Parasite *Entamoeba histolytica* through In Silico Modeling and Docking Studies

**DOI:** 10.1155/2021/9913625

**Published:** 2021-10-06

**Authors:** Mrinalini Roy, Sanket Kaushik, Anupam Jyoti, Vijay Kumar Srivastava

**Affiliations:** ^1^Amity Institute of Biotechnology, Amity University Rajasthan, Kant Kalwar, NH-11C, Jaipur-Delhi Highway, Jaipur, India; ^2^Faculty of Applied Sciences and Biotechnology, Shoolini University of Biotechnology and Management Sciences, Bajhol, Solan, Himachal Pradesh 173229, India

## Abstract

*Entamoeba histolytica* (*Eh*) is a pathogenic eukaryote that often resides silently in humans under asymptomatic stages. Upon indeterminate stimulus, it develops into fulminant amoebiasis that causes severe hepatic abscesses with 50% mortality. This neglected tropical pathogen relies massively on membrane modulation to flourish and cause disease; these modulations range from the phagocytic mode for food acquisition to a complex trogocytosis mechanism for tissue invasion. Rab GTPases form the largest branch of the Ras-like small GTPases, with a diverse set of roles across the eukaryotic kingdom. Rab GTPases are vital for the orchestration of membrane transport and the secretory pathway responsible for transporting the pathogenic effectors, such as cysteine proteases (*Eh*CPs) which help in tissue invasion. Rab GTPases thus play a crucial role in executing the cytolytic effect of *E. histolytica*. First, they interact with Gal/Nac lectins required for adhering to the host cells, and then, they assist in the secretion of *Eh*CPs. Additionally, amoebic Rab GTPases are vital for encystation because substantial vesicular trafficking is required to create dormant amoebic cysts. These cysts are the infective agent and help to spread the disease. The absence of a “bonafide” vesicular transport machinery in *Eh* and the existence of a diverse repertoire of amoebic Rab GTPases (*Eh*Rab) hint at their contribution in supporting this atypical machinery. Here, we provide insights into a pseudoRab GTPase, *Eh*RabX10, by performing physicochemical analysis, predictive 3D structure modeling, protein-protein interaction studies, and in silico molecular docking. Our group is the first one to classify *Eh*RabX10 as a pseudoRab GTPase with four nonconserved G-motifs. It possesses the basic fold of the P-loop containing nucleotide hydrolases. Through this in silico study, we provide an introduction to the characterization of the atypical *Eh*RabX10 and set the stage for future explorations into the mechanisms of nucleotide recognition, binding, and hydrolysis employed by the pseudo*Eh*Rab GTPase family.

## 1. Introduction

Membrane dynamics play an indispensable role in not only the transport of molecules through the parasite and its environment, but it is also a requisite for the host-parasite relationship. Membrane dynamics come into play at the first point of contact with the host and eventually support the creation of multiple copies of the parasite inside the host [1, 2]. Phagocytosis is a well-known phenomenon of membrane dynamics observed universally from single-celled amoebas to the complex defences of the human body. *Entamoeba histolytica*, a deadly tropical pathogen, adopts not one but two membrane modulation phenomena, namely, phagocytosis and trogocytosis, to create a severe prognosis of invasive amoebic colitis [3].

This seemingly simple organism takes more than 55,000 human lives each year and is the leading cause of death by diarrhoea in children below 5 years of age in low-income countries. It often lurks silently inside the human body until one day under the right stimulus; it manifests itself as severe amoebic colitis with a mortality rate of more than 50%. This makes it imperative for us to understand more about the pathogenesis of *E. histolytica*, focusing on the attachment, invasion, and cytolytic abilities of the pathogen; all these processes are governed by vesicular trafficking [4, 5].

The various steps of the membrane dynamics in a cell are coordinated by small GTPases that are essentially GTP/GDP molecular switches. According to their sequence, structure, and reactivity to botulinum toxin C3, they are divided into five families and subsequent subfamilies. Rab is the largest subfamily of the Ras-like GTPase superfamily. Different Rab GTPases are localized in different cellular compartments and orchestrate the vesicular trafficking seamlessly [3, 6–8]. The genome of the pathogenic amoeba *E. histolytica* has 102 annotated genes encoding a stupendous number of 91 Rab GTPases, yet the highest documented number in the available genomes of the eukaryotic kingdom. Some of these are perfect homologues to classical GTPases and others largely divergent. The vigorous and atypical endocytosis machinery observed in *E. histolytica* (*Eh*) provides support to the existence of a wide variety of Rab GTPases required to regulate this delicate machinery [9, 10].

The G-domain in the Rab GTPases facilitates shuttling between GTP- and GDP-bound stages. It belongs to the most common protein fold in the natural world, the P-loop containing fold family of nucleotide hydrolases [11]. The five motifs of this universal fold are designated G1 to G5 with highly conserved sequences—GDSGVGKS (G1/P-loop), T (G2/switch I), DTAGQ (G3/switch II), GNKCDL (G4), and SAK (G5), pivotal to the functioning of the GTPase. Previous studies have reported that 52 out of the 91 *Eh*Rab enzymes have more than 40% identity to human, yeast, and amoebic homologues. However, the remaining 39 are peculiar in their G-domain sequences and do not show substantial identity to other eukaryotic small GTPases. Thus, a separate group was created for these 39 Rab GTPases. Three of these were previously discovered (*Eh*RabA, *Eh*RabB, and *Eh*RabH) and 36 were newly identified proteins (*Eh*RabX members) [12, 13]. Surprisingly, only nine amoebic GTPases have been characterized to date and even fewer are well understood [14–18]. One cannot stress enough the importance of investigating this huge repertoire of small molecular switches in amoebic protozoans, which underpin the complex vesicular arrangements seen in *E. histolytica* and probably aid in making it a notorious pathogen [19]. It was initially believed that *E. histolytica* lacks endoplasmic reticulum and Golgi system; however, more recent studies have provided proof of the presence of an ER-Golgi system albeit not of the conventional type. The ER is distributed uniformly in the amoebic cytosol and not clustered together near the nucleus. Another unexpected observation was the continuous synthesis and movement of new proteins through the cell even after the collapse of Golgi. This indicates the presence of an atypical membrane transport system involving the diversified collection of Rab GTPases in the pathogenic amoeba [9, 20, 21].

The applications of vesicular dynamics can have a direct impact on creating therapeutics to control amoebiasis, currently managed only by the nitroimidazoles which do not come without their barrage of toxic side effects [4]. One such application is mentioned in a recent paper communicating that proanthocyanidins via manipulation of the multivesicular body (MVB) pathway act as effective antitrypanosomal compounds displaying low toxicity in humans [22]. Taking the lead from here, we decided to characterize the grossly underrepresented master membrane regulators, amoebic Rab GTPases. We selected a greatly divergent protein from the atypical *Eh*RabX family, *Eh*RabX10, and subjected it to computational biology delving into the physicochemical analysis, protein structure modeling, molecular docking, and protein-protein interaction (PPI) analysis to explore the premise of discovering the functioning of this atypical GTPase in the vesicular transport machinery of pathogenic *E. histolytica*.

## 2. Materials and Methods

### 2.1. Accession of Protein Sequence

EhRabX10 (*EHI_096440, AB197094*) protein sequence for *Entamoeba histolytica* (*Eh*) was retrieved from Amoeba DB (https://amoebadb.org/amoeba/app) and subjected to further analysis.

### 2.2. Physicochemical Characterization

The physicochemical properties of the target protein sequence were assessed on molecular weight, isoelectric point (pI), extinction coefficient, instability index, aliphatic index, and the GRAVY (grand average of hydropathicity) index using the Expasy ProtParam tool (https://web.expasy.org/protparam/) [23].

### 2.3. Sequence Alignment and Conserved Motif Identification

The Clustal W 2.0 [24] tool at the NPS server was used to obtain multiple alignments of the *Eh*RabX10 sequence with the other proteins of relevance. The identification of conserved domains was done through the NCBI Conserved Domain Database [25].

### 2.4. Structure Modeling and Validation

The information regarding the structure of EHI_096440 is not available in the RCSB PDB database [26]. Thus, we utilized homology modeling to generate a protein structure of EHI_096440 (*Eh*RabX10). Protein templates for in silico modeling of *Eh*RabX10 were obtained by running a BLAST search through the PDB, NCBI, I-TASSER, and SWISS-MODEL databases [26–28]. The sequence similarity and query coverage cut-offs were 30% and 70%, respectively. The normalised *Z*-score (template-target alignment score) and global model quality estimate (GMQE) cut-offs were 1.0 and 0.5, respectively. The templates above the cut-off values were used for homology modeling of *Eh*RabX10, through SWISS-MODEL via the Expasy web server [29]. The homology models were then analyzed for their quality via the SWISS-MODEL server. Further quality analysis was done by assessing the structural deviation (RMSD) between the template and the model, through PyMOL Molecular Graphics System (http://www.pymol.org). The final selected model of *Eh*RabX10 was subjected to validation of the structural quality using the Ramachandran plot [30] through the PDBSUM server (http://www.ebi.ac.uk/thornton-srv/databases/pdbsum/Generate.html) [31, 32].

### 2.5. Protein-Protein Interaction Study

To find out the interacting partners of our protein of interest, *Eh*RabX10 (EHI_096440), we used the STRING version 11.0 (http://string-db.org/). It generated a network view of the interacting proteins. These interacting proteins were displayed as nodes, with the node edges indicating the confidence scores. The confidence scores were generated by integrative STRING algorithms that collect and compute information from seven evidence parameters; these are experiments, text mining, cooccurrence, gene fusion, coexpression, conserved neighbourhood, and databases. The multiple coloured lines in the network map represent the seven evidence parameters supporting the interaction [33, 34].

### 2.6. Docking Studies

The proteins with high confidence interactions were subjected to molecular docking via the ClusPro server 2.0 (https://cluspro.org) [35]. None of the interacting partners of *Eh*RabX10 had their three-dimensional structure defined in the databases. Since only the PDB format is accepted as input in ClusPro, we subjected the interacting proteins to homology modeling via the SWISS-MODEL server. We performed the docking of *Eh*RabX10 with the 3D structures of the interacting partners and the algorithm generated eighty docked sets. These docked sets were based on the ClusPro PIPER algorithm energy terms: hydrophobic and electrostatic interactions, Van der Waals forces, and interatomic charges. The low-energy docked structures were subjected to rigorous qualitative and quantitative analysis via PyMOL (v.1.2r3pre; Schrodinger LLC) and Protein Interaction Calculator (http://pic.mbu.iisc.ernet.in/), respectively [36].

## 3. Results

### 3.1. Sequence-Based Investigation of *Eh*RabX10 (*EHI*_096440)

**Figure 1:**
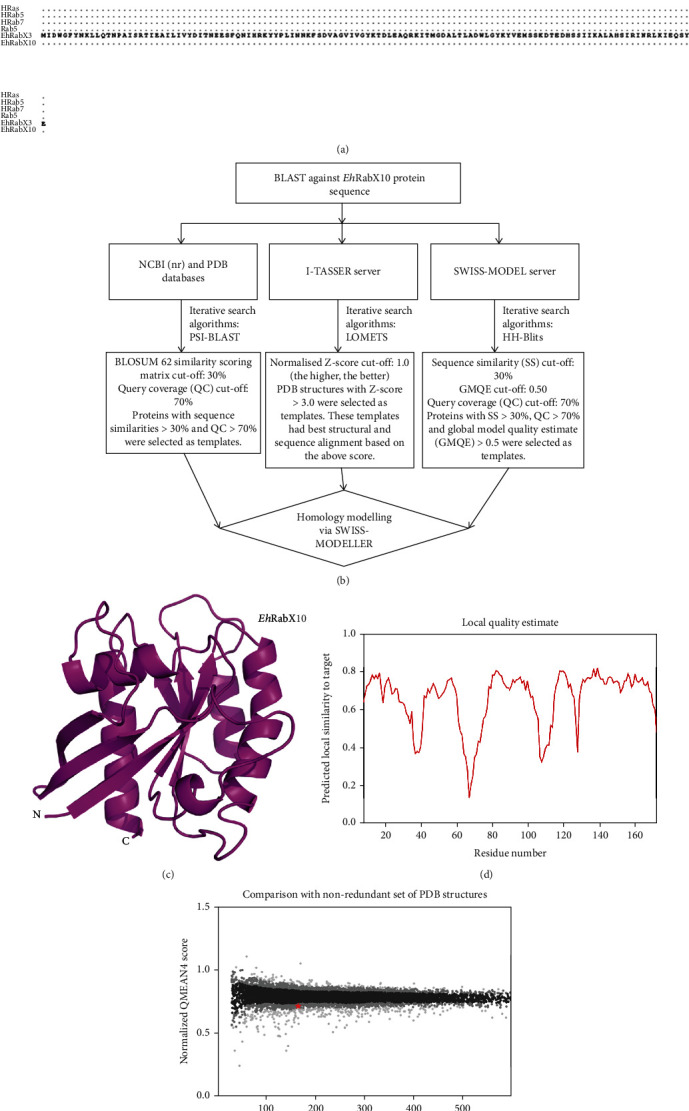
Decoding the sequence features of *Eh*RabX10 and constructing its 3D model through *in silico* methods. (a) Sequence alignment of *Eh*RabX10 with the canonical GTPases HRas (human), HRab5 (human), HRab7 (human), and Rab5 (*E. histolytica*) and the noncanonical GTPase *Eh*RabX3 (*E. histolytica*). The divergent motifs in *Eh*RabX10 are G2/switch I (absent), G3/switch II (DTQDME), G4 (TKAD), and G5 (SSQ). (b) Flowchart describing the selection of templates for homology modeling of *Eh*RabX10. (c) The modeled 3D structure of *Eh*RabX10. (d) The local quality estimate plot of the model showing the appreciable strength of local similarities between the target (*Eh*RabX10) and the template (PDB: 6O62). (e) QMEAN plot of *Eh*RabX10 is a mean of *Z*-scores that describes the “degree of nativeness” of the structural features observed in the model on a global scale. QMEAN *Z*-scores closer to zero indicate good nativeness. Our model falls in the range 1 < *Z*‐score < 2; thus, it is in agreement with structural conformations of crystal structures of similar-sized proteins.

The EHI_096440 (UniProt ID: Q5NT06) protein, commonly known as *Eh*RabX10, consists of 188 amino acid residues. The physicochemical properties of molecular mass and isoelectric point (pI) are 21.45 kDa and 5.32, respectively. The extinction coefficient is 19160 M^−1^ cm^−1^ at 280 nm, measured in water. The physicochemical indices including instability, aliphatic, and GRAVY index were calculated to be 30.63, 76.65, and −0.49, respectively. By the careful investigation of the sequence through the NCBI conserved domain database and Expasy ProtParam tool, we found that this protein has four motifs indicative of Rab GTPases, namely, G1 motif (residues 18-25), G3 motif (residues 61-66), G4 motif (residues 122-126), and G5 motif (residues 151-153). It also houses a coiled-coil domain at residues 124-144. The G1 motif (GxxxGKS/T) is conserved; however, the G2 box threonine, an extremely conserved residue in canonical Rab GTPases such as HRas [37] and HRab5 [38], is absent in *Eh*RabX10. It was also observed that three out of the four motifs (G3-G5) in *Eh*RabX10 have nonconserved sequences. The conserved sequences of these motifs are G3 (DxxGQE), G4 (NKxD), and G5 (SAK) for canonical GTPases [39] as seen in Figure 1(a); however, *Eh*RabX10 houses divergent sequences in these motifs which are G3 (DTQDME), G4 (TKAD), and G5 (SSQ).

### 3.2. Molecular Modeling of *Eh*RabX10 (EHI_096440), Proteins from *Entamoeba histolytica*

To better understand the relevance of EHI_096440, a structure was needed and the absence of a three-dimensional X-ray structure of EHI_096440 in the PDB database necessitated homology modeling of the protein. A thorough search for ideal templates was done via the servers of NCBI, PDB, I-TASSER, and SWISS-MODEL. The templates were selected based on the highest similarity to the sequence and the structural folds (secondary protein structures), predicted model quality, and percent query coverage (QC) (Figure 1(b)). The following templates scored higher than the cut-off values as mentioned in Section 2.4 and were selected for homology modeling: Rab GTPase Sec4p of *Candida albicans* (PDB ID: 6O62) [40], Ras-related protein Rab39B of *Homo sapiens* (PDB ID: 6S5F) [41], GTPase Kras isoform 2B of *Homo sapiens* (PDB ID: 4DSU) [42], GTPase Kras isoform 2B of *Homo sapiens* (PDB ID: 4DST) [42], and GTP-binding protein YPT7P of *Saccharomyces cerevisiae* (PDB ID: 1KY3) [43]. Homology models, based on all the above templates, were subjected to further scrutiny by assessing the QMEAN, GMQE (global model quality estimate), and RMSD (root mean square deviation) values (Table 1). Models with QMEAN scores closer to zero are of better quality, and models with QMEAN scores below −4.0 are rejected. Additionally, models with higher GMQE scores are considered of higher quality. Furthermore, a good model has a very low (closer to zero) root mean square deviation (RMSD) from the template.

**Figure 2:**
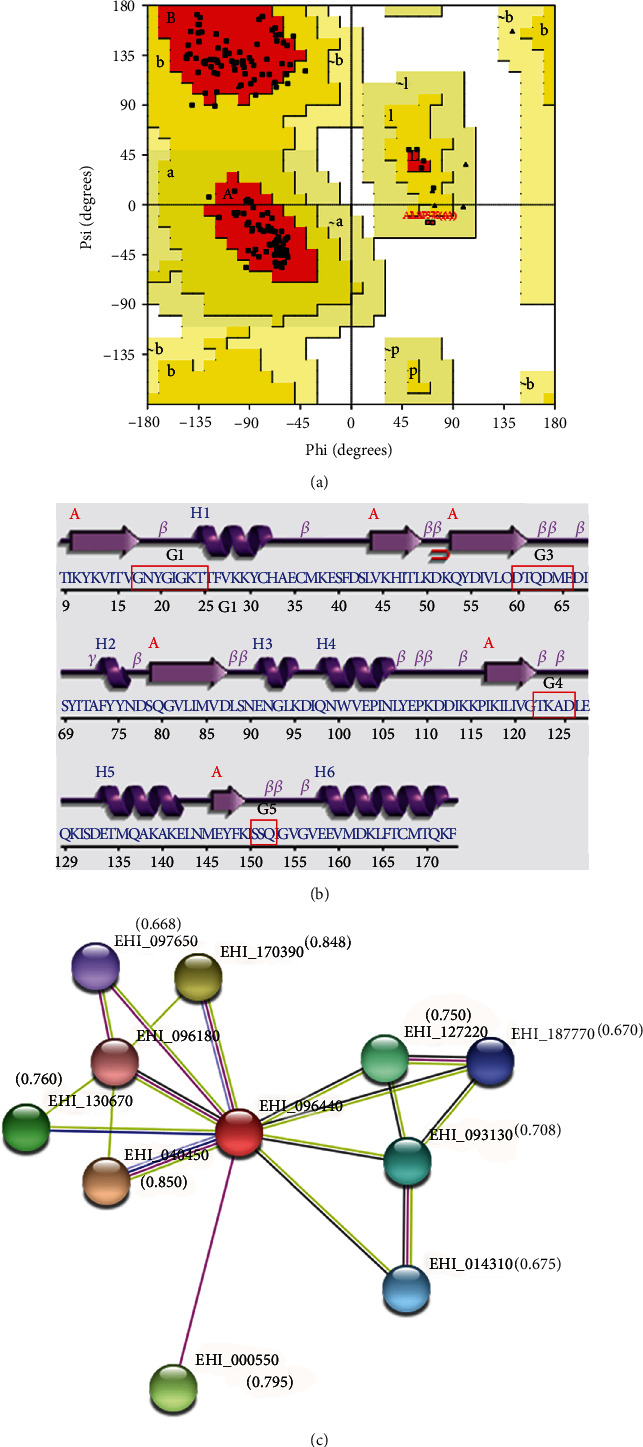
Structure quality assessment and the protein interactome of *Eh*RabX10. (a) Ramachandran plot of *Eh*RabX10 computed through PROCHECK. (b) Topology of the secondary structures of *Eh*RabX10 aligned with the FASTA sequence of *Eh*RabX10. The G-domain motifs are marked with red boxes. (c) STRING network of predicted interacting partners of EHI_096440 (*Eh*RabX10). The nodes represent the proteins, and the lines represent the functional evidence links between the proteins. The combined confidence scores are highlighted in the brackets over the nodes.

Here, we observed the highest GMQE score (0.61) and the least RMSD value (0.124 Å) for the model built on the template, Rab GTPase Sec4p of *Candida albicans* (PDB ID: 6O62). The query coverage (QC) was good (86%), covering all the crucial G-motifs of the G-domain barring only the terminal residues (1-8 and 174-188) of the *Eh*RabX10 sequence. Additionally, it also had a good model QMEAN score of −1.51 which is closer to zero (Table 1). All these factors indicate the 6O62-based *Eh*RabX10 model to be of the most credible quality [44]. This model of size 18.15 kDa was selected as final, and all further assessments were done using this model (Figures 1(c)–1(e)). The analysis of the structural quality of the *Eh*RabX10 model was done through the Ramachandran plot (Figure 2(a)). The plot computed that 90.8% residues of the *Eh*RabX10 structure lay in the most-favoured regions, 7.9% in the additionally allowed regions, 1.3% in the generously allowed regions, and none in the disallowed regions indicating the modeled structure to be of high quality when compared to the stereochemistry of protein structures experimentally decoded to date [30, 45].

The topological model generated by PROCHECK (Figure 2(b)) revealed our protein to possess the classical fold of the Rab GTPase family. This fold is composed of a six-stranded *β*-sheet with one antiparallel and five parallel strands which are interspersed by five distinct *α*-helices. In agreement with the convention, the four Rab motifs of the G-domain (G1-G5), except the G2 box (absent), are located in the loops connecting the *α*-helices and *β*-strands, and this can be observed in the modeled structure (Figure 2(b)).

### 3.3. Interactome

The STRING v11.0 server provided an interactive network (Figure 2(c)) composed of ten predicted interactions with the protein of interest, *Eh*RabX10 (EHI_096440). Each of these interacting proteins had a combined confidence score computed by combining the individual scores of each of the seven evidence parameters mentioned in Section 2.5. Indeed, all the interactions had a combined confidence score greater than 66% indicating a low chance of false positives (Table 2). We selected the protein partners with high confidence scores (>70%) for molecular docking studies (Table 3). Since none of the predicted interacting partners had a 3D structure resolved, we resorted to homology modeling and those providing the highest quality structures were used for the docking studies in ClusPro 2.0 server. Among the six, high confidence interactions, Myb-like DNA-binding domain-containing protein (EHI_000550) was not amenable to predictive modeling; however, the other five were able to produce protein structures of reliable standard (Table 2). Out of these five, we selected two proteins for further studies: the Rab family GTPase *Eh*RabC8 (EHI_170390) and the syntaxin-binding protein, *Eh*Sec1 (EHI_093130). These proteins were subjected to docking after a final structural quality check where both fared excellently with high GMQE scores of 0.62 and 0.60 of EHI_093130 and EHI_170390, respectively (Table 3). The functional links of these docked partners of *Eh*RabX10 were supported by the STRING evidence parameters of in vitro experiments, text mining, homology, and coexpression.

### 3.4. Assessment of the Docked Complexes

**Figure 3:**
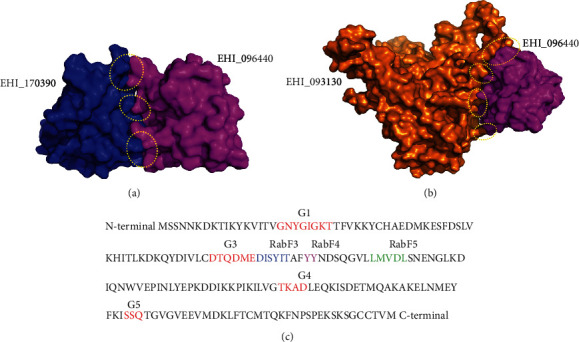
Assessment of the protein-protein interactions of *Eh*RabX10 with its partner proteins. Docked complexes of EHI_096440 (magenta) with (a) EHI_170390 (blue) and (b) EHI_093130 (orange) visualised through PyMOL. Interacting interface marked in dotted circles. (c) The complete sequence of *Eh*RabX10 displaying the diagnostic G-domain motifs and RabF regions required for the specificity of interacting effectors. The sequences are colour coded as red for the G-domain (G1-G5) motifs, blue for the RabF3 region, pink for the RabF4 region, and green for the RabF5 region. The interacting partners EHI_017390 and EHI_093130 both docked at the switch II region (G3, RabF3, and RabF4) of EHI_096440 (*Eh*RabX10).

The modeled 3D structures of interacting partners, EHI_170390 and EHI_093130, were fed into ClusPro 2.0 server as receptor proteins and EHI_096440 (*Eh*RabX10) as the ligand. The low-energy docked complexes were rendered into four groups of energy coefficients; these are balanced, electrostatically favoured, hydrophobic favoured, and Van der Waals. We assessed the top 10 complexes from each group using PyMOL and supplemented the assessment with quantitative data from Protein Interaction Calculator (PIC) server for both the receptor proteins (Figures 3(a) and 3(b)). After careful examination of ~80 docked sets, we identified the interacting residues of EHI_096440 to fall mainly in the switch II region composed of G3 box (DTQDME), RabF3 box (DISYIT), and RabF4 box (YY), for both the partners, EHI_170390 and EHI_093130 (Figure 3(c)). As established for classical Rab GTPases, the switch II region is involved in interaction with Rab effectors and regulators [11, 46]. Thus, we can speculate from the molecular docking data that this region might also serve the same function in *Eh*RabX10.

The interacting residues of EHI_170390 (*Eh*RabC8) are positioned at residue 3 Gln (N-terminus) and residues 45-79 (mainly in RabF1 and RabF3 regions) (Figure 1(a), Table 4). The interacting residues of EHI_093130 are positioned at the C-terminal (485-521) (Table 5). Singularly, we also found residues 41 Phe and 42 Asp of *Eh*RabX10 to participate in hydrophobic and ionic interactions with the partner proteins. Based on the multiple alignment data (Figure 1(a)), these two residues fall in the region annotated as RabF1 in canonical Rab GTPases but have not yet been annotated for *Eh*RabX10. This observation should be explored in future studies.

## 4. Discussion

Rab family members, though sharing the conventional G-domain of P-loop containing nucleotide hydrolases, are distinguished from the other small GTPases by virtue of the Rab family regions (RabF) flanking the G-domain motifs. Moreover, these regions are bracketed by Rab subfamily specific regions (RabSF) which provide for the specificity of the diverse Rab community across eukaryotes [46]. The discovery of these regions aided in the identification and classification of new Rab GTPases, among which lies our enzyme of interest, *Eh*RabX10, of the pathogen *Entamoeba histolytica* [12].

With nothing known about this novel GTPase, we started with the physicochemical characterization based on the amino acid sequence of *Eh*RabX10, where the instability, aliphatic, and GRAVY indices were calculated. The instability index (II) indicates the stability of a protein in a test tube. *Eh*RabX10 has an II of less than 40.0; it is stable in vitro. The aliphatic index (AI) is the relative volume occupied by the aliphatic side chains, and a high value of 76.65 signifies our protein to be thermostable. Apart from being stable, it is also essential for a protein to be pure for its study and applications, thus underlining the importance of knowing the extinction coefficient of a protein. This coefficient conveys the amount of light a protein absorbs at a certain wavelength. The extinction coefficient of *Eh*Rabx10 was 19160 M^−1^ cm^−1^ at 280 nm, measured in water, a value of importance when considering the spectrophotometric analysis of purified protein at 280 nm. The negative GRAVY index of hydropathicity defines our protein as globular and hydrophilic, which endorses the small GTPase annotation of *Eh*Rabx10.

However, an interesting contrast during sequence-based evaluation revealed the absence of the prominent G2 box threonine, vital for binding guanine nucleotide and Mg^+2^ ion in small GTPases and is the most conserved motif of the GTPase fold [37]. In addition, a massive divergence was noted in the other conserved motifs of the globular G-domain through sequence assessment. Multiple alignment of *Eh*RabX10 with classical Rab GTPases of humans (HRas [37]and HRab5 [38]) and *Entamoeba histolytica* (Rab5 [47]) and another nonclassical GTPase (*Eh*RabX3 [48]) revealed maximum deviations in the G3, G4, and G5 motifs (Figure 1(a)). The conserved residues of G3 (DxxGQE), G4 (NKxD), and G5 (SAK) are crucial for GTP/GDP state switching and hydrolysis and such significant alterations in the G-domain might affect the enzymes functioning greatly [37, 49]. We thus have labelled *Eh*RabX10 as a pseudoRab GTPase possessing noncanonical G-motifs.

**Figure 4:**
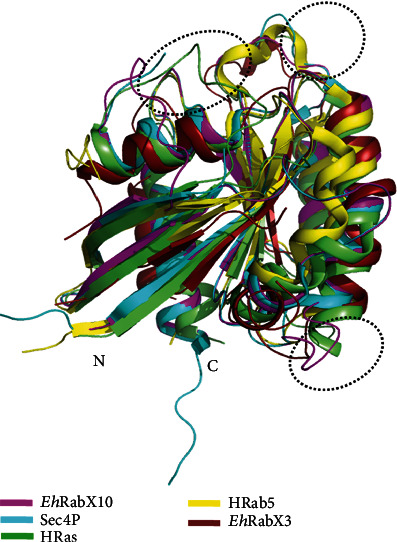
Superposition of target *Eh*RabX10 (magenta) over template Sec4p (PDB: 6O62) (cyan) and with other notable Rab GTPases: HRas (PDB ID: 6ZL3) [55] (lime green), HRab5 (PDB ID: 1TU4) [38] (yellow), and *Eh*RabX3 (PDB ID: 5C1T) [48] (raspberry) to assess the structural alignments. The dotted circles indicate the loops of maximum deviation between the structures.

Homology modeling was necessitated by the absence of the crystal structure of EHI_096440 (*Eh*RabX10). It was a rigorous process in the bid to create the best possible predictive 3D model. After iterative examination of several templates and their corresponding models, Rab GTPase Sec4p of *Candida albicans* (PDB ID: 6O62) was used as a template to model EHI_096440. The crystal structure of 6O62 protein was itself resolved at a high resolution of 1.88 Å, thus providing a detailed and reliable template for creating our model. Consequently, the predicted structure was of a high standard as mentioned in Section 3.2; none of the residues lay in the disallowed region of the Ramachandran plot, illustrating the feasibility of the phi and psi dihedral angles of all residues in our structure. Extended proof of template credibility was exhibited by the low structural deviation (RMSD 0.124 Å) between the template and the model. The model was approximately 18.15 kDa in size, which supports its annotation of a small Rab GTPase. The *Eh*RabX10 structure was also aligned with other notable Rab GTPases, among which *Eh*RabX3 was of consequence (Figure 4), being the only documented pseudoRab GTPase with its structure defined through X-ray diffraction by Srivastava et.al [48]. Quite unexpectedly, the least deviation was observed in *Eh*RabX10 alignment with HRab5 (RMSD 1.978), not *Eh*RabX3 (Table 6), and this implies that all noncanonical Rab enzymes are not identical in their structural arrangements. More research is needed to better understand the pseudoGTPase family.

In agreement with the classical Rab fold [37], *Eh*RabX10 houses six *β-*strands inside a partial shell of five *α-*helices with the loops connecting the secondary structures (Figure 1(c)). These loops contain the functional motifs (G1-G5), albeit the motifs are quite divergent as compared to the conserved sequences of conventional Rab GTPases. To explore the effects of these altered sequences on the enzyme functionality and interaction with other biomolecules, we generated an interactome of *Eh*RabX10 (EHI_096440) through the STRING v11.0 server. The interacting partners predicted with high confidence fell mostly in the category of fellow Rab GTPases and one in the syntaxin-binding protein (STXBP) family that regulate vesicle docking and fusion [50, 51] (Table 3). High-quality structures of *Eh*RabC8 (EHI_170390) and *Eh*Sec1 (EHI_093130) were built through SWISS-MODEL and were then used for docking studies in ClusPro 2.0. Experimental evidence acquired from STRING v11.0 demonstrated a strong functional link between EHI_170390 and *Eh*RabX10 (EHI_096440). It was based on affinity chromatography and tandem affinity purification assays. Coexpression evidence in the interactome showed that the STXBP partner (EHI_093130) is often coexpressed with Rab GTPases and controls the late-stage vesicular trafficking. Thus, we selected these two proteins for docking studies [50].

As described in the opening of this discussion, the RabF and RabSF regions are diagnostic for the diverse Rab GTPases found in the eukaryotic kingdom. These regions are clustered in the switch I and II regions of the GTPase [46]. In-depth tracking of residues of the protein-protein interaction complexes showed that the G3 box, RabF3, and RabF4 regions comprising the switch II loop were engaged in the docked interface of EHI_096440 (Figure 3(c)). Accordingly, it can be speculated that the diverged G-motifs may not render *Eh*RabX10 inactive and it may function as an atypical GTPase similar to the known functionally active hydrolase, *Eh*RabX3, which also lacks the G2 domain. However, the altered G4 motif in *Eh*RabX10 could considerably affect the nucleotide recognition potential of this pseudoGTPase [16, 48, 52]. A closer inspection is required to validate these hypotheses.

Previous literature has shown that the yeast homologue of Rab8, the Sec4 protein, plays a regulatory role in the late-stage vesicular secretory pathway [1]; *Eh*RabX10 is modeled using a template that is a Sec4 protein (PDB: 6O62). Additionally, *Eh*RabX10 is predicted to interact with syntaxin-binding protein of the Sec1 family (EHI_093130). These observations suggest the putative involvement of *Eh*RabX10 in the cascade of Rab-Sec signalling in the late-stage vesicular pathway [53]. There is limited knowledge about the role of amoebic Rab GTPases, except *Eh*Rab5, in the early stages of the endocytic pathway [15, 47]. In addition, there is a huge gap in understanding the orchestration of the secretion of various virulence factors such as amoebic cysteine proteases (*Eh*CPs). *E. histolytica* (*Eh*) houses a huge arsenal of *Eh*CPs, and these are the major contributors to the cytolytic potential of *E. histolytica* (*Eh*) [8, 54]. *Eh* also contains a complex and unique system of vesicle-based de novo protein trafficking that continues to function even after the collapse of the Golgi complex [20]. This compels us to explore the role of these noncanonical Rab GTPases in regulating the stages of vesicle trafficking and the *de novo* synthesis of virulence factors in the peculiar vesicular transport system of *E. histolytica* [9, 20]. The absence of these pseudoRab GTPases (*Eh*RabX family) in humans hallmarks them as targets for future exploration and justifies extensive research in discovering their mechanisms of nucleotide recognition, binding, and hydrolysis which may throw new light on the complex membrane dynamics of enteric amoebic protozoa.

## Figures and Tables

**Table 1:** Comparative analysis of the templates for homology modeling of *Eh*RabX10.

PDB ID (name)	Query coverage (%)	GMQE (global model quality estimate)	Sequence similarity (%)	Method of structure determination of template	Model QMEAN	RMSD between template and model
6O62 (Rab GTPase Sec4p, *Candida albicans*)	86	**0.61**	34	X-ray diffraction, 1.88 Å	**-1.51**	**0.124** Å
6S5F (Ras-related protein Rab39B, *Homo sapiens*)	87	0.54	34	X-ray diffraction, 1.70 Å	-2.25	0. 292 Å
4DSU (GTPase Kras isoform 2B, *Homo sapiens*)	94	0.59	32	X-ray diffraction, 1.70 Å	-1.97	2.78 Å
4DST (GTPase Kras isoform 2B, *Homo sapiens*)	94	0.58	32	X-ray diffraction, 2.3 Å	-1.21	2.078 Å
1KY3 (GTP-binding protein YPT7P, *Saccharomyces cerevisiae*)	90	0.49	35	X-ray diffraction, 1.35 Å	-3.34	0.58 Å

**Table 2:** Interactome parameters of EHI_096440 (*Eh*RabX10).

Accession no.	Annotation	Confidence score	Evidence of interaction	Amenable to homology modeling
EHI_040450	Rab family GTPase; *Eh*RabK2	0.850	Experiments, cooccurrence, text mining, homology	Yes
EHI_170390	Rab family GTPase; *Eh*RabC8	0.848	Experiments, text mining, homology	Yes
EHI_000550	Myb-like DNA-binding domain-containing protein	0.795	Experiments	No
EHI_130670	Rab family GTPase; *Eh*RabX35	0.760	Cooccurrence, text mining	Yes
EHI_127220	Synapsin, putative	0.750	Coexpression, text mining	Yes
EHI_093130	*Eh*Sec1; Syntaxin-binding protein, putative; belongs to STXBP/unc-18/SEC1 family	0.708	Coexpression, text mining	Yes
EHI_014310	Calcium-gated potassium channel protein, putative	0.675	Coexpression, text mining	No
EHI_187770	SH3 domain protein	0.670	Coexpression, text mining	Yes
EHI_097650	Rab family GTPase; *Eh*RabN2	0.668	Experiments, text mining	Yes
EHI_096180	Ras family protein	0.664	Experiments, coexpression, text mining	Yes

**Table 3:** Homology modeling of interacting partners that have high confidence interaction score.

Accession number	Annotation	Sequence similarity (%)	Query coverage (%)	GMQE (global model quality estimate)	Combined confidence score of interaction
EHI_093130	*Eh*Sec1; syntaxin-binding protein, putative; belongs to STXBP/unc-18/SEC1 family	35	**93**	**0.62**	0.708
EHI_170390	Rab family GTPase; *Eh*RabC8	39	**88**	**0.60**	0.848
EHI_040450	Rab family GTPase; *Eh*RabK2	40	80	0.54	0.850
EHI_127220	Synapsin, putative	40	93	0.70	0.750
EHI_130670	Rab family GTPase; *Eh*RabX35	39	85	0.56	0.760

**(a):** 

Position	Residue	Chain	Position	Residue	Chain
41	PHE	A	62	TRP	B
41	PHE	A	77	TYR	B
65	MET	A	45	PHE	B
65	MET	A	62	TRP	B
68	ILE	A	47	PHE	B

**(b):** 

Protein-protein side chain-side chain hydrogen bonds							
Donor				Acceptor			
Position	Chain	Residue	Atom	Position	Chain	Residue	Atom
25	A	THR	OG1	77	B	TYR	OH
38	A	LYS	NZ	72	B	ASN	OD1
67	A	ASP	OD1	3	B	GLN	OE1
67	A	ASP	OD1	3	B	GLN	OE1
3	B	GLN	OE1	67	A	ASP	OD1
3	B	GLN	OE1	67	A	ASP	OD1
58	B	LYS	NZ	67	A	ASP	OD1
58	B	LYS	NZ	67	A	ASP	OD2
77	B	TYR	OH	39	A	GLU	OE1
79	B	ARG	NH1	42	A	ASP	OD1
79	B	ARG	NH1	42	A	ASP	OD1
79	B	ARG	NH2	42	A	ASP	OD1
79	B	ARG	NH2	42	A	ASP	OD1
79	B	ARG	NH2	42	A	ASP	OD2
79	B	ARG	NH2	42	A	ASP	OD2

**(a):** 

Position	Residue	Chain	Position	Residue	Chain
20	TYR	A	495	PHE	B
20	TYR	A	496	ALA	B
20	TYR	A	499	PRO	B
37	MET	A	188	PRO	B
65	MET	A	495	PHE	B
74	PHE	A	485	PRO	B
75	TYR	A	490	LEU	B

**(b):** 

Protein-protein side chain-side chain hydrogen bonds							
Donor				Acceptor			
Position	Chain	Residue	Atom	Position	Chain	Residue	Atom
67	A	ASP	OD2	520	B	GLN	NE2
67	A	ASP	OD2	520	B	GLN	NE2
70	A	TYR	OH	520	B	GLN	OE1
75	A	TYR	OH	483	B	GLU	OE1
75	A	TYR	OH	483	B	GLU	OE2
110	A	LYS	NZ	486	B	GLU	OE1
110	A	LYS	NZ	486	B	GLU	OE2
183	B	ARG	NH2	65	A	MET	SD
183	B	ARG	NH2	65	A	MET	SD
187	B	LYS	NZ	39	A	GLU	OE1
187	B	LYS	NZ	39	A	GLU	OE2
449	B	ARG	NE	35	A	GLU	OE1
449	B	ARG	NE	35	A	GLU	OE2
449	B	ARG	NH1	35	A	GLU	OE2
449	B	ARG	NH1	35	A	GLU	OE2
453	B	LYS	NZ	39	A	GLU	OE1
478	B	ARG	NH1	42	A	ASP	OD2
478	B	ARG	NH1	42	A	ASP	OD2
478	B	ARG	NH2	42	A	ASP	OD1
478	B	ARG	NH2	42	A	ASP	OD1
478	B	ARG	NH2	42	A	ASP	OD2
478	B	ARG	NH2	42	A	ASP	OD2
520	B	GLN	NE2	67	A	ASP	OD2
520	B	GLN	NE2	67	A	ASP	OD2
520	B	GLN	OE1	70	A	TYR	OH
520	B	GLN	OE1	70	A	TYR	OH
521	B	LYS	NZ	70	A	TYR	OH

**Table 6:** Root mean square deviation (RMSD) of *Eh*RabX10 from HRas, HRab5, *Eh*RabX3, and the template Sec4p.

Name of protein	Method of structure determination	RMSD (from *Eh*RabX10 model)
Human Ras GTPase (HRas)	X-ray diffraction	3.042 Å
Human Rab GTPase (HRab5)	X-ray diffraction	1.978 Å
*Entamoeba histolytica* GTPase (EhRabX3)	X-ray diffraction	3.432 Å
*Candida albicans* Rab GTPase (Sec4p)	X-ray diffraction	**0.124 Å**

## Data Availability

Others will be able to access these data in the same manner as the authors. The authors did not have any special access privileges that others would not have.

## References

[1] Hutagalung A. H., Novick P. J. (2011). Role of Rab GTPases in membrane traffic and cell physiology. *Physiological Reviews*.

[2] Gabe Lee M.-T., Mishra A., Lambright D. G. (2009). Structural mechanisms for regulation of membrane traffic by Rab GTPases. *Traffic*.

[3] Verma K., Srivastava V. K., Datta S. (2020). Rab GTPases take centre stage in understandingEntamoeba histolyticabiology. *Small GTPases*.

[4] Rawat A., Singh P., Jyoti A., Kaushik S., Srivastava V. K. (2020). Averting transmission: a pivotal target to manage amoebiasis. *Chemical Biology & Drug Design*.

[5] Kantor M., Abrantes A., Estevez A. (2018). *Entamoeba histolytica*: updates in clinical manifestation, pathogenesis, and vaccine development. *Canadian Journal of Gastroenterology & Hepatology*.

[6] Stenmark H. (2009). Rab GTPases as coordinators of vesicle traffic. *Nature Reviews. Molecular Cell Biology*.

[7] Pfeffer S. R. (2017). Rab GTPases: master regulators that establish the secretory and endocytic pathways. *Molecular Biology of the Cell*.

[8] Rawat A., Roy M., Jyoti A., Kaushik S., Verma K., Srivastava V. K. (2021). Cysteine proteases: battling pathogenic parasitic protozoans with omnipresent enzymes. *Microbiological Research*.

[9] Perdomo D., Aït-Ammar N., Syan S., Sachse M., Jhingan G. D., Guillén N. (2015). Cellular and proteomics analysis of the endomembrane system from the unicellular *Entamoeba histolytica*. *Journal of Proteomics*.

[10] Orozco E., Betanzos A., Bañuelos C., Javier-Reyna R., García-Rivera G. (2020). Vesicular trafficking in *Entamoeba histolytica* is essential for its virulence. *Eukaryome Impact on Human Intestine Homeostasis and Mucosal Immunology*.

[11] Pylypenko O., Hammich H., Yu I. M., Houdusse A. (2018). Rab GTPases and their interacting protein partners: structural insights into Rab functional diversity. *Small GTPases*.

[12] Saito-Nakano Y., Loftus B. J., Hall N., Nozaki T. (2005). The diversity of Rab GTPases in *Entamoeba histolytica*. *Experimental Parasitology. Vol 110*.

[13] Leipe D. D., Wolf Y. I., Koonin E. V., Aravind L. (2002). Classification and evolution of P-loop GTPases and related ATPases^1^. *Journal of Molecular Biology*.

[14] Mitra B. N., Saito-Nakano Y., Nakada-Tsukui K., Sato D., Nozaki T. (2007). Rab11B small GTPase regulates secretion of cysteine proteases in the enteric protozoan *parasiteEntamoeba histolytica*. *Cellular Microbiology*.

[15] Verma K., Saito-Nakano Y., Nozaki T., Datta S. (2015). Insights into endosomal maturation of human holo-transferrin in the enteric *parasiteEntamoeba histolytica*: essential roles of Rab7A and Rab5 in biogenesis of giant early endocytic vacuoles. *Cellular Microbiology*.

[16] Chandra M., Mukherjee M., Srivastava V. K., Saito-Nakano Y., Nozaki T., Datta S. (2014). Insights into the GTP/GDP cycle of RabX3, a novel GTPase fromEntamoeba histolyticawith tandem G-domains. *Biochemistry*.

[17] Verma K., Datta S. (2017). Rab35 Participates in E. histolytica Phagocytosis. *The Journal of Biological Chemistry*.

[18] Juárez P., Sanchez-Lopez R., Stock R. P., Olvera A., Ramos M. A., Alagón A. (2001). Characterization of the _Eh_ rab8 gene, a marker of the late stages of the secretory pathway of *Entamoeba histolytica*. *Molecular and Biochemical Parasitology*.

[19] Herman E., Siegesmund M. A., Bottery M. J. (2017). Membrane Trafficking Modulation during _Entamoeba_ Encystation. *Scientific Reports*.

[20] Manning-Cela R., Marquez C., Franco E., Talamas-Rohana P., Meza I. (2003). BFA-sensitive and insensitive exocytic pathways in *Entamoeba histolytica* trophozoites: their relationship to pathogenesis. *Cellular Microbiology*.

[21] Santi-Rocca J., Smith S., Weber C. (2012). Endoplasmic reticulum stress-sensing mechanism is activated in *Entamoeba histolytica* upon treatment with nitric oxide. *PLoS One*.

[22] Kubata B. K., Nagamune K., Murakami N. (2005). Kola acuminata proanthocyanidins: a class of anti-trypanosomal compounds effective against *Trypanosoma brucei*. *International Journal for Parasitology*.

[23] Gasteiger E., Hoogland C., Gattiker A. (2005). Protein identification and analysis tools on the ExPASy server. *The Proteomics Protocols Handbook*.

[24] Larkin M. A., Blackshields G., Brown N. P. (2007). Clustal W and Clustal X version 2.0. *Bioinformatics*.

[25] Marchler-Bauer A., Derbyshire M. K., Gonzales N. R. (2015). CDD: NCBI’s conserved domain database. *Nucleic Acids Research*.

[26] Rose P. W., Beran B., Bi C. (2011). The RCSB Protein Data Bank: redesigned web site and web services. *Nucleic Acids Research*.

[27] Camacho C., Coulouris G., Avagyan V. (2009). BLAST+: architecture and applications. *BMC Bioinformatics*.

[28] Steinegger M., Meier M., Mirdita M., Vöhringer H., Haunsberger S. J., Söding J. (2019). HH-suite3 for fast remote homology detection and deep protein annotation. *BMC Bioinformatics*.

[29] Waterhouse A., Bertoni M., Bienert S. (2018). SWISS-MODEL: homology modelling of protein structures and complexes. *Nucleic Acids Research*.

[30] Ramachandran G. N., Ramakrishnan C., Sasisekharan V. (1963). Stereochemistry of polypeptide chain configurations. *Journal of Molecular Biology*.

[31] Laskowski R. A. (2001). PDBsum: summaries and analyses of PDB structures. *Nucleic Acids Research*.

[32] Laskowski R. A., MacArthur M. W., Moss D. S., Thornton J. M. (1993). PROCHECK: a program to check the stereochemical quality of protein structures. *Journal of Applied Crystallography*.

[33] Szklarczyk D., Gable A. L., Lyon D. (2019). STRING v11: protein-protein association networks with increased coverage, supporting functional discovery in genome-wide experimental datasets. *Nucleic Acids Research*.

[34] von Mering C., Jensen L. J., Snel B. (2004). STRING: known and predicted protein-protein associations, integrated and transferred across organisms. *Nucleic Acids Research*.

[35] Kozakov D., Hall D. R., Xia B. (2017). The ClusPro web server for protein-protein docking. *Nature Protocols*.

[36] Tina K. G., Bhadra R., Srinivasan N. (2007). PIC: protein interactions calculator. *Nucleic Acids Research*.

[37] Colicelli J. (2004). Human RAS superfamily proteins and related GTPases. *Science's STKE*.

[38] Zhu G., Zhai P., Liu J., Terzyan S., Li G., Zhang X. C. (2004). Structural basis of Rab5-Rabaptin5 interaction in endocytosis. *Nature Structural & Molecular Biology*.

[39] Nakada-Tsukui K., Saito-Nakano Y., Husain A., Nozaki T. (2010). Conservation and function of Rab small GTPases in *Entamoeba*: Annotation of *E. invadens* Rab and its use for the understanding of *Entamoeba* biology. *Experimental Parasitology*.

[40] Stogios P. J., Skarina T., Di Leo R., Savchenko A., Center for Structural Genomics of Infectious Diseases (CSGID) (2019). *Crystal structure of Sec4p, a Rab family GTPase from Candida albicans*.

[41] Diaz-Saez L., Jung S., von Delft F. (2019). *Structure of the human RAB39B in complex with GMPPNP*.

[42] Maurer T., Garrenton L. S., Oh A. (2012). Small-molecule ligands bind to a distinct pocket in Ras and inhibit SOS-mediated nucleotide exchange activity. *Proceedings of the National Academy of Sciences*.

[43] Constantinescu A. T., Rak A., Alexandrov K., Esters H., Goody R. S., Scheidig A. J. (2002). Rab-subfamily-specific regions of Ypt7p are structurally different from other RabGTPases. *Structure*.

[44] Benkert P., Biasini M., Schwede T. (2011). Toward the estimation of the absolute quality of individual protein structure models. *Bioinformatics*.

[45] Morris A. L., MacArthur M. W., Hutchinson E. G., Thornton J. M. (1992). Stereochemical quality of protein structure coordinates. *Proteins: Structure, Function, and Genetics*.

[46] Pereira-Leal J. B., Seabra M. C. (2000). The mammalian Rab family of small GTPases: definition of family and subfamily sequence motifs suggests a mechanism for functional specificity in the Ras superfamily^1^. *Journal of Molecular Biology*.

[47] Srivastava V. K., Kaushik S., Jyoti A. (2019). A comparativein silicoanalysis of Rab5 proteins from pathogenic species to find its role in the pathogenesis. *Journal of Molecular Recognition*.

[48] Srivastava V. K., Chandra M., Saito-Nakano Y., Nozaki T., Datta S. (2016). Crystal Structure Analysis of Wild Type and Fast Hydrolyzing Mutant of *Eh* RabX3, a Tandem Ras Superfamily GTPase from *Entamoeba histolytica*. *Journal of Molecular Biology*.

[49] White M. A., Nicolette C., Minden A. (1995). Multiple ras functions can contribute to mammalian cell transformation. *Cell*.

[50] Jorgačevski J., Potokar M., Grilc S. (2011). Munc18-1 tuning of vesicle merger and fusion pore properties. *The Journal of Neuroscience*.

[51] Jorgačevski J., Kreft M., Zorec R. (2017). Exocytotic pore in a SNARE. *Oncotarget*.

[52] Kumar Srivastava V., Chandra M., Datta S. (2014). Crystallization and preliminary X-ray analysis of RabX3, a tandem GTPase from *Entamoeba histolytica*. *Acta Crystallographica Section F Structural Biology Communications*.

[53] Batra S., Pancholi P., Roy M. (2021). Exploring insights of syntaxin superfamily proteins fromEntamoeba histolytica: a prospective simulation,protein‐proteininteraction, and docking study. *Journal of Molecular Recognition*.

[54] Espinosa-Cantellano M., Marti’nez A., Marti’nez-Palomo M. Pathogenesis of intestinal amebiasis: from molecules to disease. Vol 13. http://cmr.asm.org/.

[55] Kessler D., Bergner A., Böttcher J. (2020). Drugging all RAS isoforms with one pocket. *Future Medicinal Chemistry*.

